# Analysis of GPS/EGNOS Positioning Quality Using Different Ionospheric Models in UAV Navigation

**DOI:** 10.3390/s23031112

**Published:** 2023-01-18

**Authors:** Grzegorz Grunwald, Adam Ciećko, Tomasz Kozakiewicz, Kamil Krasuski

**Affiliations:** 1 Faculty of Geoengineering, University of Warmia and Mazury in Olsztyn, 10-720 Olsztyn, Poland; 2 Institute of Navigation, Polish Air Force University, 08-521 Dęblin, Poland

**Keywords:** GPS/EGNOS, satellite-based augmentation system (SBAS), unmanned aerial vehicle (UAV), Klobuchar and NeQuick

## Abstract

Unmanned aerial vehicles (UAVs) have become very popular tools for geoinformation acquisition in recent years. They have also been applied in many other areas of life. Their navigation is highly dependent on global navigation satellite systems (GNSS). The European Geostationary Navigation Overlay Service (EGNOS) is intended to support GNSSs during positioning, mainly for aeronautical applications. The research presented in this paper concerns the analysis of the positioning quality of a modified GPS/EGNOS algorithm. The calculations focus on the source of ionospheric delay data as well as on the aspect of smoothing code observations with phase measurements. The modifications to the algorithm concerned the application of different ionospheric models for position calculation. Consideration was given to the EGNOS ionospheric model, the Klobuchar model applied to the GPS system, the Klobuchar model applied to the BeiDou system, and the NeQuick model applied to the Galileo system. The effect of removing ionospherical corrections from GPS/EGNOS positioning on the results of the determination of positioning quality was also analysed. The results showed that the original EGNOS ionospheric model maintains the best accuracy results and a better correlation between horizontal and vertical results than the other models examined. The additional use of phase-smoothing of code observations resulted in maximum horizontal errors of approximately 1.3 m and vertical errors of approximately 2.2 m. It should be noted that the results obtained have local characteristics related to the area of north-eastern Poland.

## 1. Introduction

In recent years, there has been a rapid increase in the number of unmanned aircraft systems (UAS), their technical complexity, and sophistication [1]. Unmanned aviation technological developments are currently moving at a much faster pace than for manned aviation. It is estimated that the European drone market alone will be worth EUR 10 billion annually by 2035 and over EUR 15 billion annually by 2050 [2]. This has resulted in a high level of interest among users, which is opening up new opportunities for drone use in various sectors of the market. Drones are currently being used not only for recreational and sporting purposes but are also becoming part of professional data acquisition systems for various purposes, mainly due to their relatively low production and purchase costs and ease of use. In the past, the development of unmanned aerial vehicle (UAV) systems and platforms was primarily motivated by military goals and applications. Unmanned inspection, surveillance, reconnaissance, and mapping of inimical areas were the primary military aims. Some of the first civilian drone users were surveyors, who were already using air raids (using classic aircraft) to acquire geo-information data. A key element for the acquisition of reliable and accurate data is the UAV navigation system. An accurate real-time positioning and navigation system is important for both the execution of the UAV mission (flight along the designed survey profiles) and for the high accuracy of the georeferenced image data. The higher the accuracy, the better the quality of the final product in the form of a map, digital surface model (DSM), or digital elevation model (DEM).

For commercial drones, a GNSS is most commonly used for navigation. The majority of commercially available UAVs are equipped with single-frequency GNSS receivers [3,4], thereby providing a positioning accuracy of 8–13 m [5] through a standard positioning service (SPS). For single-frequency receivers, remote sensing data and their quality is dependent on ground control points (GCPs). They are mainly surveyed by ground-based methods, resulting in measurement data not being fully obtained remotely. It is also possible to carry out fully remote UAV measurements without the need for GCPs. For this purpose, dual-frequency solutions using real-time kinematic (RTK) technology and providing positioning accuracies of a few centimetres are used, but these solutions are relatively expensive and used by a small number of users [6,7]. Previous research shows that RTK positioning using drones may be less accurate due to the lack of information on the phase centre of the GNSS antenna [8]. In addition, the non-linearity of the movement of a drone in the air is one of the elements that can negatively affect theoretical assumptions about RTK positioning [9]. A very interesting and promising solution is the use of satellite-based augmentation systems (SBAS) for navigation and UAV data acquisition [10,11]. An SBAS is used for single-frequency receivers; its use is cheap to apply and increases positioning accuracy to the level of 1–2 metres, while at the same time providing positioning integrity and informing the user in real time about a decrease in positioning accuracy during the performed mission. The accuracy of the acquired geoinformation data using SBAS is admittedly not at the level of single centimetres, but it is significantly better than the accuracy of the SPS service and can satisfy many professional users without the need to set up and measure GCPs.

SBASs were originally designed to enhance the performance of standard GNSS positioning in aviation. SBASs improve the positioning accuracy by providing corrections for the largest error sources. Therefore, SBASs broadcast real-time correction products such as the ionosphere model, satellite orbits, and clock errors which can provide reliable positioning services with high level of integrity which is essential for safety-critical transport applications in various domains; in particular, the service is compliant with aviation requirements for approaches with vertical guidance (APV-I) and Category I precision approaches. Several interoperable SBASs have been or are being implemented around the world due to the benefits they provide. The already operational systems include: the American Wide Area Augmentation System (WAAS), the European Geostationary Navigation Overlay Service (EGNOS), the Japanese Satellite Augmentation System (MSAS), and the Indian GPS Aided Geo Augmented Navigation (GAGAN). The systems under implementation include the Russian System for Differential Corrections and Monitoring (SDCM), Chinese BeiDou SBAS (BDSBAS), and Australian Southern Positioning Augmentation Network (SouthPAN). There are also systems which are still planned under feasibility studies. These include: the South American and Caribbean Soluciόn de Aumentaciόn para Caribe, Centro y Sudamérica (SACCSA), African A-SBAS, and South Korean Korea Augmentation Satellite System (KASS) [12].

The EGNOS system is a European SBAS based on geostationary satellites used for the transmission of differential corrections. EGNOS is an international project whose construction and operation are jointly supervised by the European Commission, European Space Agency (ESA), and Eurocontrol. Today, EGNOS augments only GPS using the L1 (1575.42 MHz) coarse/acquisition (C/A) civilian signal function by providing correction data and integrity information to improve positioning, navigation, and timing services over Europe. In the future, EGNOS will augment both GPS and Galileo, using L1 and L5 (1176.45 MHz) frequencies. The basic scheme is to use a set of monitoring stations (at very well-known positions) to receive navigation signals from core GNSS constellations that will be processed in order to obtain some estimations of the errors that are also applicable to users. Once these estimations have been computed, they are transmitted in the form of “differential corrections” by means of GEO satellites.

EGNOS provides three services:

- Open Service (OS), freely available to any user.
- Safety of Life (SoL) Service, that provides the most stringent level of signal-in-space performance to all Safety of Life user communities.
- EGNOS Data Access Service (EDAS) for users who require enhanced performance for commercial and professional use.

The main objective of the EGNOS OS is to improve the achievable positioning ac-curacy by correcting several error sources affecting GPS signals. The corrections transmitted by EGNOS contribute to mitigate the ranging error sources related to satellite clocks, satellite position, and ionospheric effects. EGNOS can also detect distortions affecting the signals transmitted by GPS and prevent users from tracking unhealthy or misleading signals. The main objective of the EGNOS SoL service is to support civil aviation operations down to localiser performance with vertical guidance (LPV) minima. EDAS is the EGNOS terrestrial data service which offers ground-based access to EGNOS data in real time and also in a historical FTP archive to authorised users [13].

The subject of drones and their applications is currently very popular in the literature globally. Items on the history, basics of drone construction, fundamentals of aerodynamics, applications, and general rules and regulations for the operation and use of drones are very popular among readers [14,15]. It should be noted that this topic is subject to constant development and modification. Even experienced users need to keep up to date with new possibilities, applications, and above all, changing regulations. Since the early 2000s, countries have gradually established national legal frameworks concerning UAVs. Although all UAV regulations have one common goal—minimising the risks to other airspace users and to both people and property on the ground—they are different in different countries and change very often. Another popular topic related to UAVs is their use in mapping and photogrammetry. Following a typical photogrammetric workflow with the use of UAVs, 3D results such as digital surface or terrain models, contours, textured 3D models, vector information, etc. can be produced, even on large areas [16,17,18]. A very important element related to drones is the navigation system. This topic is very popular among a number of researchers. Various navigation methods have been proposed and they can be mainly divided into three categories: satellite navigation, inertial navigation, and vision-based navigation. UAV navigation using GNSSs is the classical method in which single-frequency receivers are most commonly used [19,20]. A popular and important topic is navigation in an environment where a GNSS is difficult to access or unavailable [21]. In this case, inertial systems can be used, which are often combined with GNSS technology [22,23]. One can also use the vision-based navigation which proves to be a primary and promising research direction of autonomous navigation with the rapid development of computer vision [24,25].

The topic of using SBASs in UAV navigation is relatively new. In previous works, researchers have, among other things, used SBASs to improve positioning accuracy and reliability [11], determined the integrity of EGNOS positioning for UAV technology [26], used SBASs in UAV technology to support Search and Rescue (SAR) systems [27], examined ground surface deformation in complex landslide areas [28] or used an integrated SBAS-InSar system for landslide detection [29]. In the above-mentioned articles, the authors did not manipulate with the ionosphere model used for SBAS positioning. In the article [10], the authors decided to combine the EGNOS ionosphere model and SDCM ionosphere model. However, no publications were found in which different ionosphere models were used in SBAS-assisted UAV positioning, so this topic seems to be up-to-date and interesting.

The use of SBASs in aviation is related to compliance with existing guidelines. However, it is technically possible to modify the GNSS/SBAS positioning algorithm based on the components of pseudorange corrections and the smoothing of code observations. The research conducted so far has not been related to these aspects. Drone flights are of a different nature to those performed by aeroplanes due to, among other things, the flight range and different flight dynamics. Therefore, it seems reasonable to search for an optimal UAV positioning solution based on EGNOS.

## 2. Materials and Methods

### 2.1. Ionospheric Delay

In aeronautical GPS/EGNOS applications, according to Radio Technical Committee for Aeronautics [30], it is possible to use the ionospheric model of the original EGNOS and the Klobuchar model known from GPS autonomous positioning. The original EGNOS model, like the models associated with other SBASs, is a real time model that, with an interval of 5 min, attempts to take into account the influence of the ionosphere on positioning [31,32,33,34,35]. To determine the influence of the ionosphere on GPS/EGNOS positioning, according to the guidelines presented in RTCA (2013), it is necessary to define the ionospheric pierce point (IPP), which is the intersection between the theoretical ionospheric layer at 350 km above the Earth’s surface and the line between the receiver and the satellite [36]. Ionospheric corrections are transmitted by EGNOS geostationary satellites for each point located on the virtual grid at a height of 350 km above the Earth’s surface. Using a suitable interpolation algorithm, the delay value for a given IPP can be determined [30]. For moderate geodetic latitudes, grid points are spaced every 5 degrees of latitude and 5 degrees of longitude, and for high latitudes the resolution is 30 degrees. The slant delay associated with the EGNOS model recommended for use in precision landing approach procedures is determined according to the formula:

(1) $${\mathrm{IC}}_{i}=-F_{\mathrm{pp}}\tau_{\mathrm{vpp}}\left(\lambda_{\mathrm{pp}},\phi_{\mathrm{pp}}\right)$$

where: 

$F_{\mathrm{pp}}$—obliquity factor, 

$\tau_{\mathrm{vpp}}$—interpolated vertical delay at the IPP, 

$\lambda_{\mathrm{pp}},\phi_{\mathrm{pp}}$—coordinates of IPP.

$F_{\mathrm{pp}}$ and $\tau_{\mathrm{vpp}}$ can be determined from the formulas:

(2) $$F_{\mathrm{pp}}={\left[1-{\left(\frac{R_{e}\cos E}{R_{e}+h_{I}}\right)}^{2}\right]}^{-\frac{1}{2}}$$

(3) $$\tau_{\mathrm{vpp}}=\overset{4}{\underset{i\;=1}{\sum }}W_{i}\left(x_{\mathrm{pp}},y_{\mathrm{pp}}\right)\tau_{\mathrm{vi}}$$

where: 

$R_{e}$—radius of the Earth, 

E—elevation of the satellite, 

$h_{I}$—the altitude at which the highest electron density occurs (350 km), 

$\tau_{\mathrm{vi}}$—vertical delays for 4 grid points, transmitted by EGNOS, $W_{i}\left(x_{\mathrm{pp}},y_{\mathrm{pp}}\right)$—weighting function.

The Klobuchar model used in GPS is based on the Bent model [37]. Due to its simplicity and easy implementation, it has found application in positioning with single-frequency satellite receivers [38,39,40]. Its disadvantage is its low resistance to rapid ionospheric changes significantly affecting positioning results, since it is typically updated once per day. The values of the coefficients transmitted in the navigational broadcast message are determined by the average solar 10.7 cm flux value and the day of the year. It is defined as a single-layer ionospheric model (SLM) because it uses a theoretical layer of the ionosphere at 350 km above the Earth’s surface to calculate the delay. The Klobuchar model generates different delay values for daytime and night. Its efficiency is dependent on solar activity and user location. It is estimated that this model is able to eliminate up to 50% of the influence of the ionosphere on satellite positioning [40].

The Klobuchar ionospheric model associated with the BeiDou system is based on the determination of coefficients with a 2 h interval on the basis of locally distributed BeiDou regional stations. A layer of the ionosphere 375 km above the Earth’s surface is used in the calculation. In contrast to the GPS model, the Klobuchar BeiDou model operates using geographic instead of geomagnetic coordinates. The vertical ionospheric delay of Klobuchar can be determined using the formula [41,42,43]:

(4) $$I\left(t\right)=N+P\cdot cos\left(\frac{2\pi \left(t-14\right)}{T}\right)$$

where: 

*I*(*t*)—vertical ionosphere delay, 

*t*—local time of the ionospheric pierce point, 

*N*—night-time constant, *P*—the amplitude of the cosine term (determined on the basis of coefficients broadcasted by satellites), 

*T*—period of the cosine term (determined on the basis of coefficients broadcasted by satellites).

Obtaining slant delay values is possible through the use of a mapping function:

(5) $$MF\left(e\right)=1.0+16.0\cdot {\left(0.53-e\right)}^{3}$$

where: 

*e*—satellite elevation (given in semi-circles).

The NeQuick model was developed by the Aeronomy and Radiopropagation Laboratory of The Abdus Salam International Centre for Theoretical Physics (ICTP) in Trieste and the Institute for Geophysics, Astrophysics and Meteorology (IGAM) of the University of Graz. Initially, this model was used for EGNOS analysis [44]. Under the name NeQuick G, an algorithm was developed for positioning using the European Galileo system [45,46,47]. According to preliminary analyses, the model was found to be able to eliminate up to 70% of the ionospheric delay under nominal measurement conditions [48]. Using the *a_i_*_0_, *a_i_*_1_, and *a_i_*_2_ coefficients, updated at least once a day and transmitted similarly to the GPS system in the navigation message, the effective ionisation level (*Az*) can be determined using the formula:

(6) $$Az=a_{i0}+a_{i1}\cdot MODIP+a_{i2}\cdot {\left(MODIP\right)}^{2}$$

where: 

*MODIP*—the Modified Dip Latitude at the location of the user receiver.

Further computational steps for determining the ionospheric delay based on the Nequick G model are explained in [49]. Finally, the slant total electron content along the path is determined, which is converted to a slant delay based on the formula:

(7) $$I_{g}=\frac{40.3}{f^{2}}\int_{sat.}^{rec.}N_{e}ds=\frac{40.3}{f^{2}}sTEC$$

where: 

*I_g_*—the group delay, 

*f*—frequency, 

*N_e_*—the electron density,

*sTEC*—the Slant Total Electron Content.

### 2.2. Smoothing of Code Observations

The smoothing of pseudoranges with phase observers is intended to reduce the noise level [50]. According to the Radio Technical Committee for Aeronautics [30], SBAS-related corrections should be applied after smoothing the pseudorange carrier. The smoothing algorithm should be restarted each time the phase observation is affected by a cycle slip. This is conducted by implementing the formula according to the Hatch filter [50]:

(8) $$PR_{k}^{S}=\alpha PR_{k-1}^{C}+\left(1-\alpha \right)⟦PR_{k-1}^{S}+\frac{\lambda }{2\pi }(PR_{k}^{P}-PR_{k-1}^{P})⟧$$

(9) $$\alpha =\frac{dt}{T}$$

where: 

$PR_{k}^{S}$—smoothed pseudorange,

$PR_{k-1}^{C}$—code pseudorange in the *k*−1 epoch,

$PR_{k-1}^{S}$—smoothed pseudorange in the *k*−1 epoch,

$PR_{k}^{P}$—phase pseudorange in the *k* epoch,

λ—wavelength of GPS carrier frequency at L1,

T—smoothing constant,

dt—sampling interval.

## 3. Results

**Figure 1:**
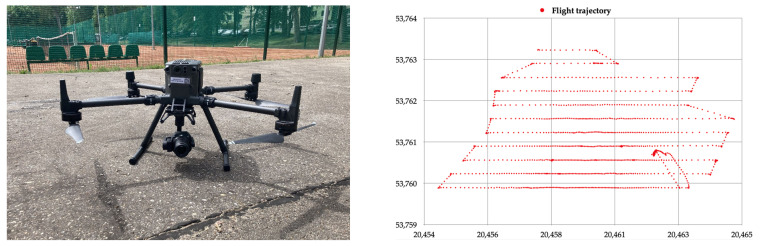
The DJI Matrice 300 RTK used to collect GNSS data (**left**) and the recorded UAV flight trajectory (**right**).

A field survey was carried out on 15 March 2022 using a DJI Matrice 300 RTK drone (Figure 1), which allows GNSS raw data to be recorded using the built-in satellite receiver. GNSS data were recorded during a flight lasting approximately 20 min. The work resulted in observation files in the standardised RINEX format with a data logging interval of 1 s. Using GLAB software and in-house scripts, calculations were made for GPS/EGNOS positioning by modifying the source data with ionospheric corrections applied to the positioning algorithm. The tests were carried out during a calm state of the ionosphere. The Kp value reflecting the electromagnetic field strength on the day the field test was carried out ranged between 1 and 3. This speaks in favour of the low activity of the ionosphere. Positioning quality results were investigated using the EGNOS ionospheric standard model, the Klobuchar GPS model, the Klobuchar BeiDou model, the NeQuick model, and a variant with no ionospheric corrections. According to the recommendations of [30], code positioning used in aviation should be smoothed with phase observations. To this end, the above variants related to ionospheric correction were analysed based on the three variants involving the smoothing of code measurements with phase observations.

The smoothing window is the multiplication of the number of samples taken in the calculation and the measurement data interval (1 s). According to the aviation recommendations, a GNSS satellite should be removed from the GNSS/SBAS solution if it has not reached steady-state, which is defined as the time for continuous smoothing of code observations with phase measurements. This is related to restrictive aeronautical applications. In the UAV experiments carried out, steady-state was given in two variants: variant one steady-state was 5 s and variant two steady-state was 60 s.

**Figure 2:**
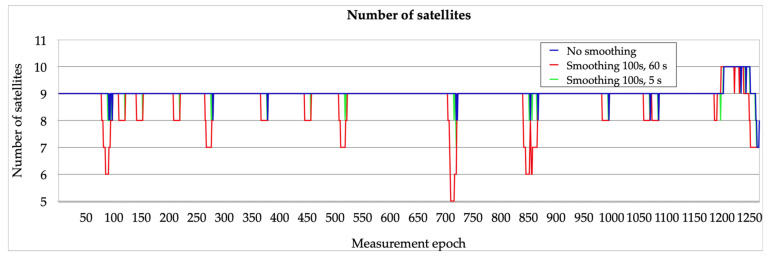
Number of satellites used in positioning in the no smoothing, smoothing 100 s, 5 s, and smoothing 100 s, 60 s variants.

**Figure 3:**
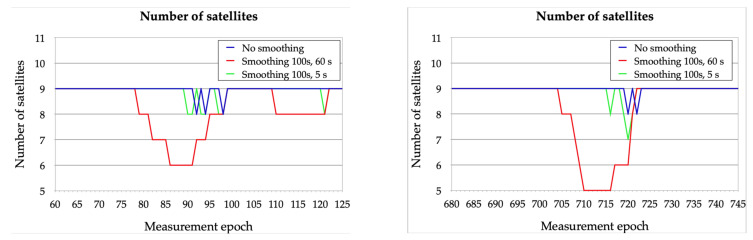

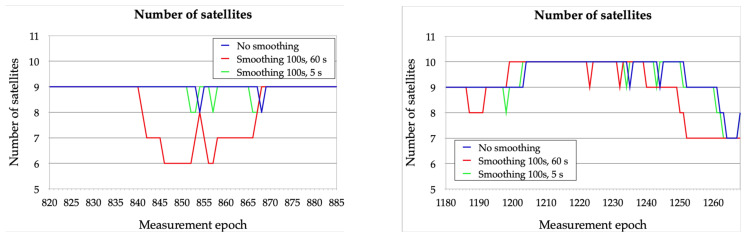
Selected sections showing the number of satellites used in positioning in the no smoothing, smoothing 100 s, 5 s, and smoothing 100 s, 60 s variants.

The first stage of the research was to analyse the number of satellites used in positioning in the different variants of no smoothing, smoothing 100 s, 5 s, and smoothing 100 s, 60 s (Figure 2). The 100 s, 5 s variant indicates a smoothing window of 100 s and a steady state of 5 s, while the 100 s, 60 s variant indicates a smoothing window of 100 s and a steady state of 60 s. This number remains constant when using different sources of ionospheric corrections. Figure 3 provides a detailed depiction of the changes in the number of satellites used in the different positioning variants. It should be noted that the 100 s, 60 s smoothing variant differs the most from the other two. The fact that a given satellite adopted for positioning in this variant must be available for at least 60 s influences the occurrence of differences in the number of satellites in comparison with the other variants. Differences in the number of satellites used for positioning between the no smoothing and 100 s, 5 s smoothing variant last less than a few seconds. For the no smoothing and smoothing 100 s, 5 s variants, the number of satellites used in the solution varies between 7 and 10. For the smoothing 100 s, 60 s variant, the range is between 5 and 10.

**Figure 4:**
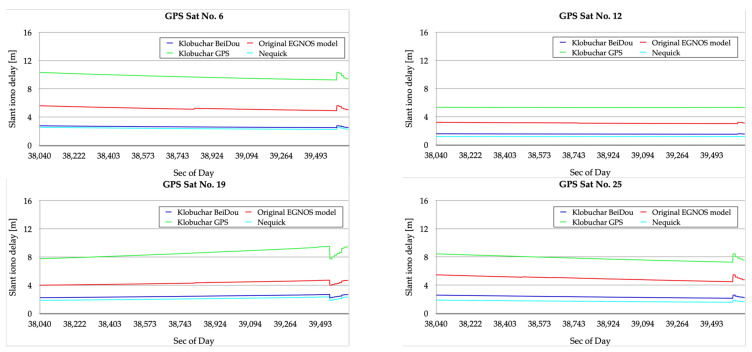
Ionospheric delay values for selected satellites associated with GPS/EGNOS positioning in different variants of the ionospheric model used: Klobuchar BeiDoU, Klobuchar GPS, NeQuick, and original EGNOS model.

The next stage of the analyses includes the characteristics of the ionospheric delay values applied in the individual variants associated with the ionospheric models. The characteristics presented in Figure 4 include the values of the ionospheric correction associated with selected GPS satellites, i.e., PRN 6, 12, 19, and 25 for the Klobuchar BeiDou model, Klobuchar GPS model, NeQuick model, and the original EGNOS model. It is noteworthy that the delay values associated with the Klobuchar BeiDou model are similar to those found for the NeQuick variant, oscillating around 2–3 m. In the case of the Klobuchar GPS model, these values vary from around 6 m to around 10 m. In the case of the original EGNOS model, the ionospheric delay values can be placed between those obtained for the NeQuick and Klobuchar BeiDou models and those obtained for the Klobuchar GPS model. They range from about 3 m to about 6 m. 

**Figure 5:**
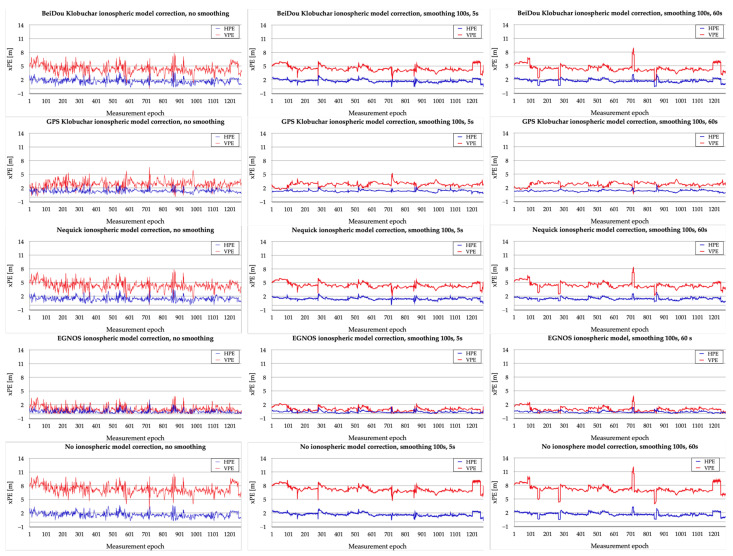
HPE and VPE for different variants related to ionospheric delay and phase smoothing of code observations.

In the next stage, the effect of smoothing the code observations with phase measurements on the accuracy of horizontal and vertical positioning was investigated. Horizontal positioning error (HPE) and vertical positioning error (VPE) values were calculated on the basis of reference positions calculated in relative positioning post-processing mode using reference stations. Figure 5 shows the results of a study of GPS/EGNOS positioning accuracy based on different smoothing variants. The greater stability of the horizontal and vertical accuracies obtained for the variants using smoothing is clearly visible. In addition, it is noticeable that the largest vertical positioning errors are achieved for the 100 s, 60 s smoothing variant. For the three variants based on the original EGNOS ionospheric model, the horizontal positioning accuracy is close to the vertical one. 

## 4. Discussion

The numerical values associated with the horizontal and vertical accuracy analysis are shown in Table 1.

The results presented in Table 1 show the lowest maximum horizontal positioning errors for the applied EGNOS standard ionospheric model and the 100 s, 60 s smoothing variant; however, similar results were obtained for the EGNOS ionospheric model variant and the 100 s, 5 s smoothing variant (1.32 m and 1.50 m were obtained, respectively). The highest horizontal maximum errors were obtained for the no smoothing variant and the applied Klobuchar BeiDou ionospheric model (3.85 m), which are close to the no smoothing variant without using any ionospheric model (3.76 m). The horizontal standard deviation obtains the best results for the used EGNOS model and 100 s, 60 s smoothing variant (0.17 m); a slightly worse result was obtained for the EGNOS and GPS Klobuchar model variants with 100 s, 5 s smoothing (0.19 cm). The worst horizontal standard deviation is observed for no smoothing calculations associated with the variants: the no ionospheric correction and Klobuchar BeiDou model (0.50 m and 0.52 m, respectively). The values of the lowest mean horizontal positioning errors were obtained for the EGNOS model variants with 100 s, 60 s smoothing and with 100 s, 5 s smoothing (0.34 m and 0.36 m, respectively), with the worst performance being seen in the Klobuchar BeiDou model variant, obtaining values of approximately 1.70 m in the variants with smoothing and in the variant without smoothing.

The lowest vertical positioning errors, which at the same time differ significantly from the other results, were obtained in the case of using the EGNOS model and smoothing of observations in the variant 100 s, 5 s, with maximum errors up to 2.22 m. The highest maximum vertical positioning errors were obtained for no ionospheric corrections and 100 s, 60 s smoothing. The most favourable values of vertical standard deviation and vertical mean errors were obtained for the EGNOS model variant 100 s, 5 s (VPE_st.dev. = 0.45 m, VPE_mean = 0.95 m). The worst vertical results were obtained for the variant without ionospheric corrections and without smoothing (VPE_max = 10.59 m, VPE_st.dev. = 0.97 m, VPE_mean = 7.20 m).

This work shows how important the ionosphere model is for the quality of UAV positioning. In previous works related to the use of SBASs in UAV navigation [10,11,26], the authors obtained similar results; however, they did not modify the ionospheric model in the solution. A very interesting approach in UAV positioning may be the use of the RTK technique [6,7,8,9]; this solution provides better accuracy, but it is much more expensive and limited to a small number of users. The obtained results highlight a good correlation between horizontal and vertical errors in GPS/EGNOS positioning when using the original EGNOS ionospheric model. This is extremely important for applications related to aerial navigation, including with UAVs. Up to now, studies carried out in this field were usually related to a fixed point position [51], rather than with kinematic measurements on the Earth’s surface [52]. Studies carried out using the UAV have made it possible to simulate measurement conditions similar to those associated with general aviation navigation.

## 5. Conclusions

The research was carried out under conditions of a calm ionospheric state in north-eastern Poland. The influence of the selection of the ionospheric model source on the positioning with the use of unmanned aerial vehicles was analysed. The use of the original EGNOS ionospheric model yielded the lowest values of horizontal and vertical positioning errors, which are also close to each other. The Klobuchar model, used in GPS, gave satisfactory results, confirming that it can be used in cases where the EGNOS model is not available. The Klobuchar model dedicated to the BeiDou system and NeQuick gave worse horizontal and vertical results than the previously mentioned models, but was able to offset the negative impact of the ionosphere on airborne navigation to some extent. Smoothing code observations with phase measurements improves the horizontal and vertical positioning error values. However, for short-duration flights, a non-optimal selection of steady-state can negatively affect horizontal and vertical positioning error values. 

The results achieved for EGNOS ionospheric model are very satisfactory. The horizontal mean accuracy in the range of one foot and vertical in the range of three feet can satisfy most UAV users, including professionals creating DTMs and DEMs. In the future, it is planned to perform further research in which the use of different ionospheric models in UAV navigation will be tested in disturbed ionospheric conditions. Other modifications of the positioning model will also be taken into consideration. The conducted research work can be used to improve the quality of UAV positioning for different professional applications.

## Figures and Tables

**Table 1:** Numerical results of accuracy analysis related to horizontal and vertical positioning errors.

		No Smooth.	Smooth. 100 s, 5 s	Smooth. 100 s, 60 s			No Smooth.	Smooth. 100 s, 5 s	Smooth. 100 s, 60 s
BeiDou Klob.	HPE_max	3.85	2.90	3.14	BeiDou Klob.	VPE_max	7.86	6.19	8.88
	HPE_st.dev.	0.50	0.32	0.37		VPE_st.dev.	0.92	0.62	0.80
	HPE_mean	1.70	1.73	1.70		VPE_mean	4.41	4.45	4.43
GPS Klob.	HPE_max	2.94	2.27	2.71	GPS Klob.	VPE_max	6.58	5.30	3.87
	HPE_st.dev.	0.35	0.19	0.18		VPE_st.dev.	0.78	0.44	0.42
	HPE_mean	1.35	1.34	1.33		VPE_mean	2.82	2.78	2.77
NeQuick	HPE_max	2.62	2.57	2.88	NeQuick	VPE_max	7.87	5.91	8.33
	HPE_st.dev.	0.44	0.26	0.27		VPE_st.dev.	0.88	0.55	0.68
	HPE_mean	1.42	1.43	1.42		VPE_mean	4.37	4.41	4.40
EGNOS	HPE_max	3.01	1.50	1.32	EGNOS	VPE_max	3.85	2.22	3.84
	HPE_st.dev.	0.35	0.19	0.17		VPE_st.dev.	0.66	0.45	0.49
	HPE_mean	0.50	0.36	0.34		VPE_mean	1.02	0.95	0.96
No corr.	HPE_max	3.76	2.90	3.27	No corr.	VPE_max	10.59	9.16	12.03
	HPE_st.dev.	0.52	0.35	0.40		VPE_st.dev.	0.97	0.69	0.91
	HPE_mean	1.66	1.68	1.66		VPE_mean	7.20	7.24	7.21

## Data Availability

The data presented in this study are available on request from the corresponding author.
